# Involvement of Raft Aggregates Enriched in Fas/CD95 Death-Inducing Signaling Complex in the Antileukemic Action of Edelfosine in Jurkat Cells

**DOI:** 10.1371/journal.pone.0005044

**Published:** 2009-04-07

**Authors:** Consuelo Gajate, Fernando Gonzalez-Camacho, Faustino Mollinedo

**Affiliations:** 1 Instituto de Biología Molecular y Celular del Cáncer, Centro de Investigación del Cáncer, Consejo Superior de Investigaciones Científicas (C.S.I.C.) – Universidad de Salamanca, Campus Miguel de Unamuno, Salamanca, Spain; 2 Unidad de Investigación, Hospital Universitario de Salamanca, Campus Miguel de Unamuno, Salamanca, Spain; Cleveland Clinic, United States of America

## Abstract

**Background:**

Recent evidence suggests that co-clustering of Fas/CD95 death receptor and lipid rafts plays a major role in death receptor-mediated apoptosis.

**Methodology/Principal Findings:**

By a combination of genetic, biochemical, and ultrastructural approaches, we provide here compelling evidence for the involvement of lipid raft aggregates containing recruited Fas/CD95 death receptor, Fas-associated death domain-containing protein (FADD), and procaspase-8 in the induction of apoptosis in human T-cell leukemia Jurkat cells by the antitumor drug edelfosine, the prototype compound of a promising family of synthetic antitumor lipids named as synthetic alkyl-lysophospholipid analogues. Co-immunoprecipitation assays revealed that edelfosine induced the generation of the so-called death-inducing signaling complex (DISC), made up of Fas/CD95, FADD, and procaspase-8, in lipid rafts. Electron microscopy analyses allowed to visualize the formation of raft clusters and their co-localization with DISC components Fas/CD95, FADD, and procaspase-8 following edelfosine treatment of Jurkat cells. Silencing of Fas/CD95 by RNA interference, transfection with a FADD dominant-negative mutant that blocks Fas/CD95 signaling, and specific inhibition of caspase-8 prevented the apoptotic response triggered by edelfosine, hence demonstrating the functional role of DISC in drug-induced apoptosis. By using radioactive labeled edelfosine and a fluorescent analogue, we found that edelfosine accumulated in lipid rafts, forming edelfosine-rich membrane raft clusters in Jurkat leukemic T-cells. Disruption of these membrane raft domains abrogated drug uptake and drug-induced DISC assembly and apoptosis. Thus, edelfosine uptake into lipid rafts was critical for the onset of both co-aggregation of DISC in membrane rafts and subsequent apoptotic cell death.

**Conclusions/Significance:**

This work shows the involvement of DISC clusters in lipid raft aggregates as a supramolecular and physical entity responsible for the induction of apoptosis in leukemic cells by the antitumor drug edelfosine. Our data set a novel framework and paradigm in leukemia therapy, as well as in death receptor-mediated apoptosis.

## Introduction

In the last few years, a growing amount of evidence suggests that apoptosis induced by Fas/CD95 death receptor is mediated by the formation of Fas/CD95 aggregates in lipid rafts [1]–[7]. Clustering of death receptor Fas/CD95 can be achieved not only by interaction with its natural ligand FasL/CD95L, but through non-physiological agents independently of its ligand [1], [4], [8], providing a new framework for novel therapeutic interventions [6]. This ligand-independent activation of Fas/CD95 has a great potential therapeutic utility as it avoids the toxic side effects derived from the use of FasL/CD95L and agonistic anti-Fas/CD95 antibodies *in vivo*, that lead to a fatal hepatic damage with symptoms similar to fulminant hepatitis [9], [10].

Edelfosine (ET-18-OCH_3_, 1-*O*-octadecyl-2-*O*-methyl-*rac*-glycero-3-phosphocholine) is the prototypic compound of a promising family of synthetic antitumor lipids, collectively named as synthetic alkyl-lysophospholipid analogues [11], which induce selective apoptosis in tumor cells while sparing normal cells [12]. Edelfosine acts through activation of the apoptotic machinery in cancer cells, involving death receptors, caspase activation, JNK/c-Jun signaling and mitochondria [1], [4], [13]–[18], and therefore this apoptosis-targeted drug could be appropriate for diseases where apoptosis is altered. Edelfosine was the first antitumor drug reported to promote an apoptotic response through FasL/CD95L-independent activation of Fas/CD95 by its recruitment in lipid rafts [1], linking for the first time membrane rafts and Fas/CD95-mediated apoptosis in cancer chemotherapy. The proapoptotic capacity of edelfosine was higher against cancer cells derived from blood malignancies than from solid tumors [19].

Stimulation of Fas/CD95 results in receptor aggregation and recruitment of the adapter molecule Fas-associated death domain-containing protein (FADD), through interaction between its own death domain and the clustered receptor death domains. FADD, in turn, contains a death effector domain that binds to an analogous domain repeated in tandem within the zymogen form of procaspase-8, forming the so-called death-inducing signaling complex (DISC), made up of Fas/CD95, FADD and procaspase-8 [20], which drives cells to apoptosis.

Despite previous findings that suggest a role of Fas/CD95 and lipid rafts in cancer chemotherapy [1], [4]–[7], [21], compelling evidence and visualization of the involvement of DISC-enriched raft clusters in cancer treatment is still lacking. Here, by using genetic, biochemical and ultrastructural approaches, we demonstrate the formation and role of DISC co-clustering in membrane rafts during edelfosine-induced apoptosis, setting up a new framework in leukemia therapy.

## Results

### Biochemical and ultrastructural evidence for the formation of DISC–enriched raft clusters in Jurkat cells treated with edelfosine

**Figure 1:**
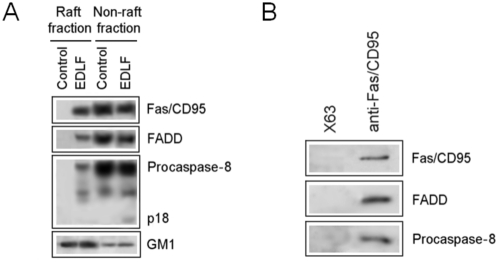
DISC formation in lipid rafts following Jurkat cell incubation with edelfosine. (A) Untreated control Jurkat cells (Control) and Jurkat cells treated with 10 µM edelfosine (EDLF) for 9 h were analyzed for lipid raft isolation on a discontinuous sucrose density gradient. Raft and non-raft fractions were analyzed by Western blotting for the indicated proteins using specific antibodies. The migration positions of the 55-kDa procaspase-8 as well as of the cleavage product p18 are denoted. Location of GM1-containing lipid rafts was determined using CTx B subunit conjugated to horseradish peroxidase. (B) Fas/CD95 was immunoprecipitated from the raft fraction of edelfosine-treated Jurkat cells. Immunoprecipitates were subjected to SDS-PAGE and immunoblotted with Fas/CD95, FADD- and procaspase-8 specific antibodies, respectively. Raft fraction was also immunoprecipitated with P3X63 (X63) myeloma supernatant as a negative control. Experiments shown are representative of three performed.

We have previously reported that edelfosine induced co-aggregates of rafts and Fas/CD95 in leukemia Jurkat T-cells and multiple myeloma cells [1], [4], [7]. The proapoptotic complex DISC was generated upon multiple myeloma cell treatment with edelfosine [7]. Here, we extend these results by finding that Fas/CD95 as well as downstream signaling molecules FADD and procaspase-8 were translocated into membrane rafts (Figure 1A), forming the proapoptotic complex DISC in lipid rafts (Figure 1B), upon edelfosine treatment of Jurkat leukemic T-cells. In contrast, co-immunoprecipitation assays conducted in untreated Jurkat cells rendered no DISC formation (data not shown). Lipid rafts were identified using cholera toxin (CTx) B subunit conjugated to horseradish peroxidase that binds to the oligosaccharide portion of ganglioside GM1 [22], [23], mainly found in lipid rafts [24].

**Figure 2:**
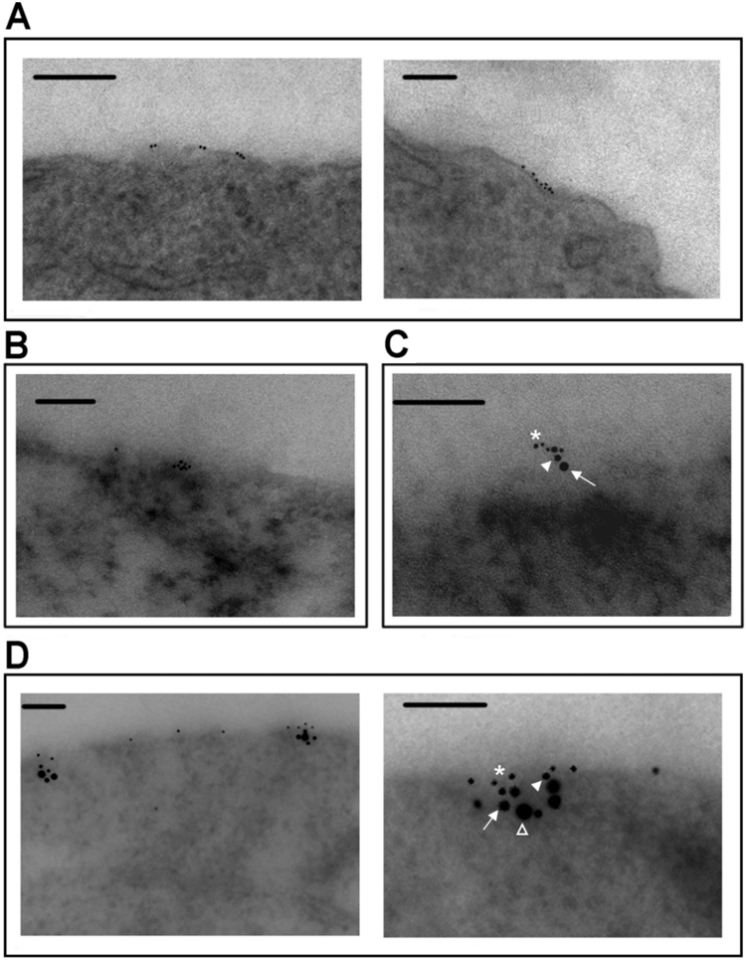
Ultrastructural localization of DISC embedded in lipid rafts during edelfosine treatment of leukemic cells. (A–D) Electron microscopy images of Jurkat cells treated with 10 µM edelfosine (EDLF) for 9 h. Sections of edelfosine-treated cells were labeled with the raft marker GM1 using CTx B subunit (6-nm gold) alone (A), or in combination with anti-Fas/CD95 antibody (10-nm gold) (B). Drug-treated cells were also labeled with the raft marker GM1 using CTx B subunit (6-nm gold) (asterisk), anti-Fas/CD95 antibody (10-nm) (closed arrowhead), anti-FADD antibody (15-nm) (arrow) (C), and anti-procaspase-8 antibody (20-nm) (open arrowhead) (D). Lipid rafts are labeled on the external face of the membrane, whereas DISC components are located in the internal face of raft-enriched membrane domains. Bar, 400 nm.

To visualize and further define the co-clustering of DISC and membrane rafts following edelfosine incubation, we used electron microscopy. This technique preserved the ultrastructural integrity of the membrane and allowed immunolabeling for DISC constituents. In untreated Jurkat cells, we only detected very disperse and weak staining of rafts and Fas/CD95 at the plasma membrane without co-localization between both labels (data not shown). However, edelfosine treatment of Jurkat cells induced clusters of lipid rafts, identified through CTx B subunit binding, at the cell surface (Figure 2A). Fas/CD95 was located embedded in plasma membrane rafts upon edelfosine treatment (Figure 2B), thus indicating the translocation and recruitment of Fas/CD95 death receptor into membrane rafts at the cell surface. Following drug treatment and using immunogold electron microscopy, FADD was immunolocalized together with Fas/CD95 and rafts, facing the cytoplasmic side of the membrane (Figure 2C). Furthermore, procaspase-8 was also localized in the same region in edelfosine-treated cells, forming DISC aggregates in discrete zones of the plasma membrane enriched in lipid rafts after drug-treated Jurkat cells were labeled with specific antibodies to each DISC component and with CTx B subunit to identify membrane rafts (Figure 2D). The presence of multiple gold particles, labeling rafts (Figure 2A–2D) and DISC components (Figure 2B–2D), suggests a high local molecule concentration of DISC constituents in raft platforms, in keeping with the formation of DISC-rich raft clusters. Our data visualize for the first time the formation of co-clusters of DISC in lipid rafts in cancer chemotherapy, and further demonstrate the role of membrane rafts as scaffolds to concentrate Fas/CD95 and downstream signaling molecules in small and specialized areas of the cell surface following edelfosine treatment.

### Functional role of Fas/CD95, FADD, and caspase-8 in drug-induced apoptosis in Jurkat cells

**Figure 3:**
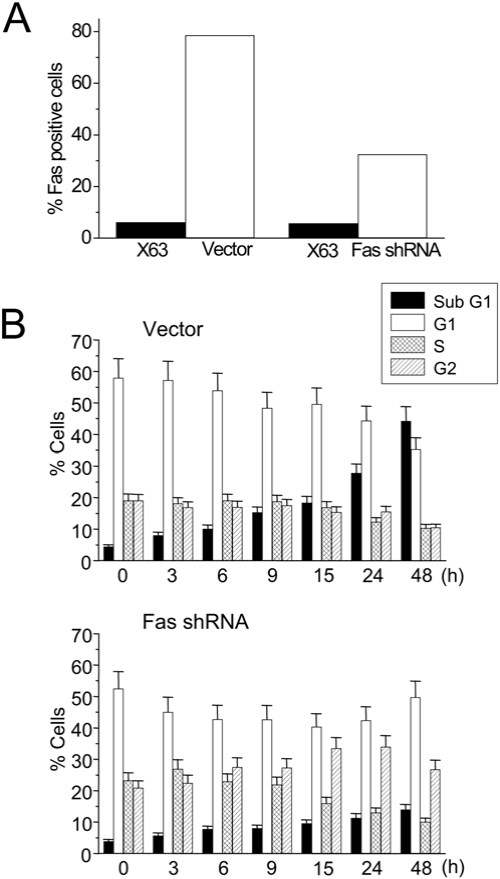
Edelfosine-induced apoptosis in Jurkat cells is mediated by Fas/CD95. (A) Cells were transfected with Fas/CD95 shRNA (target sequence 1, Materials and Methods) (Fas shRNA) or neomycin-resistant scrambled sequence control vector (Vector), and the percentage of Fas/CD95 positive cells was assessed by flow cytometry using P3X63 (X63) myeloma supernatant as a negative control. (B) Jurkat cells transfected with neomycin-resistant scrambled sequence control vector (Vector) or Fas/CD95 shRNA (target sequence 1, Materials and Methods) (Fas shRNA) were treated with 10 µM edelfosine-induced apoptosis for the indicated incubation times and the proportion of cells in each phase of the cell cycle was quantitated by fluorescence flow cytometry. Cells in the sub-G_1_ region represent apoptotic cells. Untreated control cells were run in parallel. Data are shown as means±SE of four independent experiments.

To investigate the role of DISC in edelfosine-induced apoptosis, we inhibited the endogenous expression of Fas/CD95 in Jurkat cells by RNA interference using short hairpin RNA (shRNA). This Fas/CD95 silencing resulted in a significant loss of Fas/CD95 protein expression (Figure 3A) and inhibition of edelfosine-induced apoptosis (Figure 3B). Transfection of Jurkat cells with one of the four target sequences used for Fas/CD95 silencing (target sequence 1 in the Materials and Methods section) led to clones with about 65% Fas/CD95 silencing (Figure 3A). This downregulation of Fas/CD95 protein expression was further assessed by Western blotting and by mean fluorescence intensity measurements in flow cytometry analysis (data not shown). This partial Fas/CD95 silencing led to about 70% inhibition in edelfosine-induced apoptosis after 48-h drug incubation (Figure 3B). Apoptosis rates were determined by measuring the percentage of cells at the sub-G_1_ region in cell cycle analysis (Figure 3B). Analysis of the distinct cell cycle phases showed that Fas/CD95 silencing promoted a slight G_2_/M arrest following edelfosine treatment (Figure 3B). Cell transfection with a control vector containing a scrambled sequence did not affect either Fas/CD95 expression (Figure 3A) or drug-induced apoptosis (Figure 3B), and no changes were detected in the distinct cell cycle phases before the triggering of apoptosis (Figure 3B). Likewise, additional Fas/CD95 shRNA vectors that did not silence Fas/CD95 expression were without effect on the apoptotic rate rendered by edelfosine (data not shown). Cells transfected with control vector behaved similarly to intact nontransfected Jurkat cells, regarding either Fas/CD95 protein expression or drug-induced apoptosis (data not shown).

**Figure 4 pone-0005044-g004:**
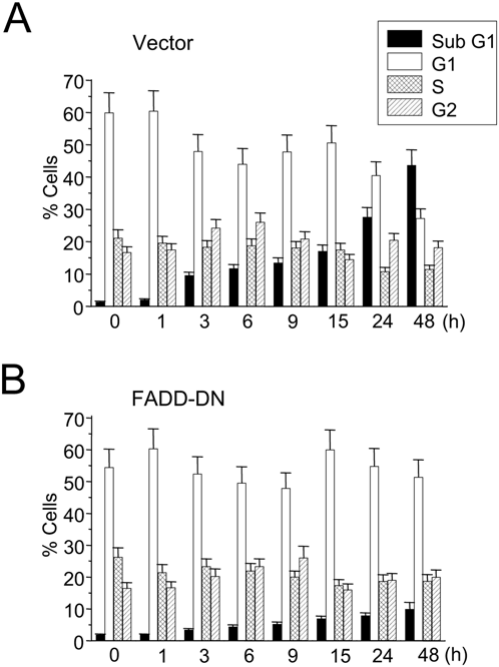
Edelfosine-induced apoptosis in Jurkat cells is mediated by FADD. Cells, stably transfected with control pcDNA3 empty vector (Vector) (A) or FADD-DN (B), were treated with 10 µM edelfosine for the indicated incubation times, and the proportion of cells in each phase of the cell cycle was quantified by flow cytometry. Cells in the sub-G_1_ region represent apoptotic cells. Untreated cells were run in parallel. Data are shown as means±SE of four independent experiments.

Using stable transfection in Jurkat cells with a dominant-negative form of the FADD adapter protein (FADD-DN), which lacks the death effector domain and prevents death receptor signaling [25], we found that blockade of Fas/CD95 downstream signaling abrogated edelfosine-induced apoptosis (about 80% inhibition after 48-h drug incubation) (Figure 4A and 4B). Conversely, transfection with a control pcDNA3 empty vector did not affect edelfosine-induced apoptosis (Figure 4A). Cells transfected with pcDNA3 control vector behaved similarly to intact nontransfected Jurkat cells regarding drug-induced apoptosis (data not shown).

**Figure 5 pone-0005044-g005:**
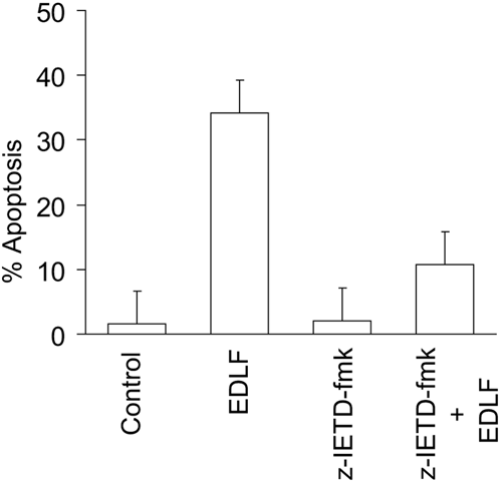
Edelfosine-induced apoptosis in Jurkat cells is mediated by caspase-8. Cells were preincubated without or with 50 µM of z-IETD-fmk for 1 h, and then incubated in the absence or presence of 10 µM edelfosine (EDLF) for 24 h, and analyzed by flow cytometry to evaluate apoptosis. Untreated control cells were run in parallel. Data are shown as means±SE of three independent experiments.

Furthermore, the cell-permeable specific caspase-8 inhibitor z-IETD-fmk (*N*-benzyloxycarbonyl-Ile-Glu-Thr-Asp-fluoromethyl ketone) strongly inhibited (about 70% inhibition) edelfosine-induced apoptosis (Figure 5).

**Figure 6:**
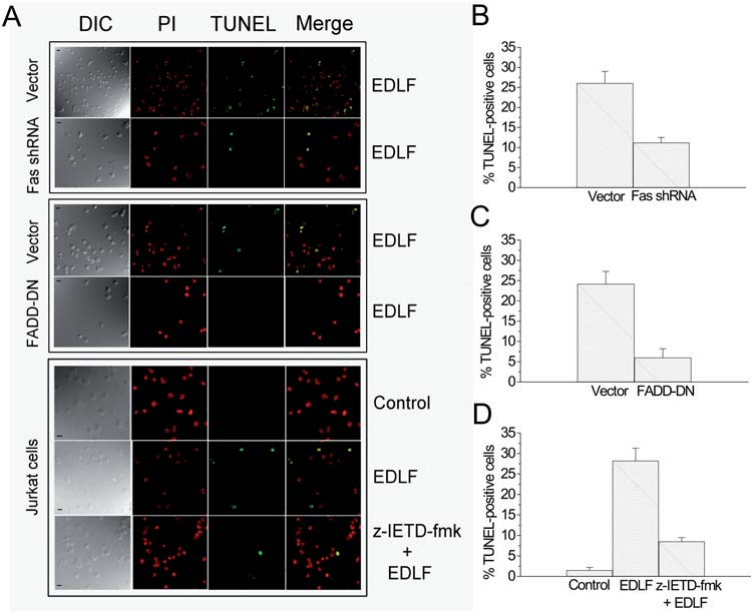
Involvement of DISC constituents in edelfosine-induced apoptosis as assessed by TUNEL assay. (A) Jurkat cells transfected with neomycin-resistant scrambled sequence control vector (Vector) or Fas/CD95 shRNA (target sequence 1, Materials and Methods) (Fas shRNA) (upper panel), and with control pcDNA3 empty vector (Vector) or FADD-DN (middle panel), were treated with 10 µM edelfosine (EDLF)-induced apoptosis for 20 h, and analyzed by confocal microscopy for differential interference contrast (DIC), propidium iodide (PI) staining and TUNEL assay. Merging of PI and TUNEL panels (Merge) shows the apoptotic nuclei in yellow. Jurkat cells were also untreated (Control), treated with 10 µM edelfosine for 20 h (EDLF), or preincubated with 50 µM of z-IETD-fmk for 1 h followed by incubation in the presence of 10 µM edelfosine for 20 h (z-IETD-fmk+EDLF), and then analyzed by confocal microscopy for DIC, PI staining and TUNEL assay as above (lower panel). Data shown are representative of four independent experiments. Bar, 10 µm. (B–D) Histograms indicate the percentage of TUNEL-positive cells, as an estimate of cells undergoing apoptosis, following the experimental conditions shown in A (upper, middle and lower panels). For each experiment at least 120 cells were analyzed. Data are shown as means±SE of four independent experiments.

The above results show that silencing or inhibition of the three major components of DISC prevents the apoptotic response induced by edelfosine in Jurkat cells as assessed by cell cycle analysis. In order to further confirm these conclusions, we analyzed apoptosis by the terminal deoxynucleotidyl transferase-mediated dUTP nick-end labeling (TUNEL) technique [26] as an *in situ* method for detecting the 3′-OH ends of DNA exposed during the internucleosomal cleavage that occurs during apoptosis (Figure 6). Labeling the 3′-OH ends, generated by DNA fragmentation, through incorporation of fluoresecin-12-dUTP allowed visualization of apoptotic cells. In addition, cells were permeabilized and stained with propidium iodide to visualized all nuclei from both non-apoptotic and apoptotic cells in red, while TUNEL-positive cells were stained in green. Silencing of Fas/CD95 by RNA interference (Figure 6A and 6B), constitutive expression of FADD-DN (Figure 6A and 6C), and inhibition of caspase-8 with z-IETD-fmk (Figure 6A and 6D) strongly inhibited edelfosine-induced apoptosis, as assessed by TUNEL analysis. The apoptotic rate, measured by this TUNEL technique, of untreated cells or Jurkat cells treated only with the caspase-8 inhibitor z-IETD-fmk, run in parallel, was less than 3% in all cases (data not shown). Similar apoptosis rates were obtained using cell cycle (hypodiploidy) and TUNEL analyses (Figures 3–6).

Taken together, we found that targeting each of the three components of DISC precludes the induction of apoptosis by the alkyl-lysophospholipid analogue edelfosine. These results strongly indicate that DISC regulates edelfosine-induced apoptosis in leukemic cells.

### Accumulation of edelfosine in lipid rafts and raft requirement for drug uptake and apoptosis

**Figure 7:**
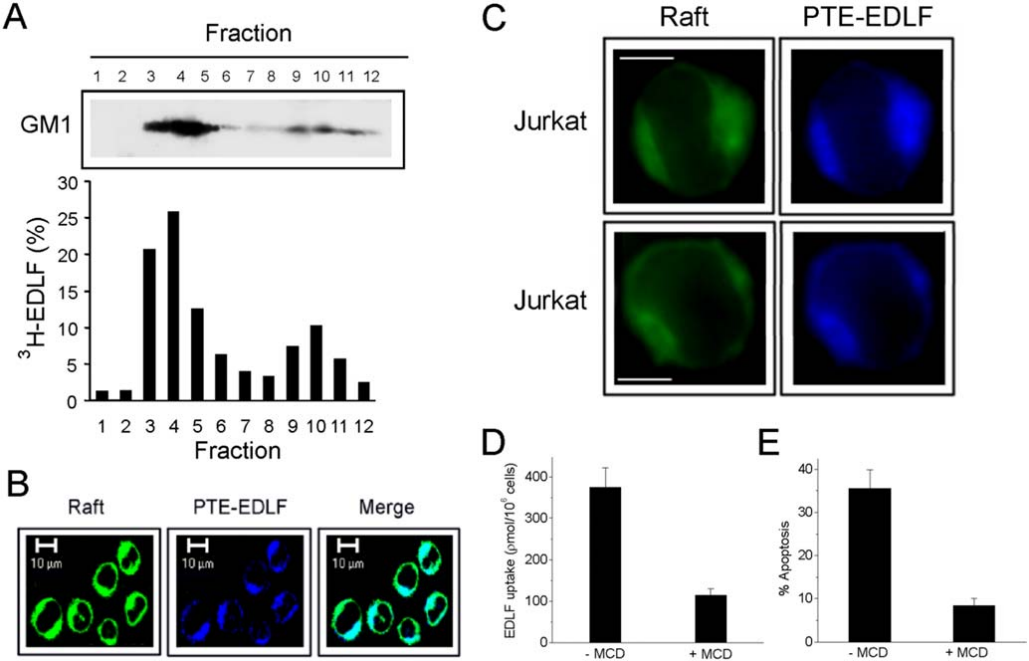
Edelfosine accumulation in lipid rafts. (A) Jurkat cells treated with 10 µM [^3^H]edelfosine for 9 h were lysed in 1% Triton and fractionated by centrifugation on a discontinuous sucrose density gradient. An equal volume of each collected fraction was subjected to SDS-PAGE and counted for radioactivity. The distribution patterns of GM1-containing rafts (upper panel) (fractions 3–5) and [^3^H]edelfosine (EDLF) (lower panel) over the gradient fractions are shown. Data are representative of three separate experiments. (B) Jurkat cells were incubated with 10 µM PTE-edelfosine (PTE-EDLF, blue fluorescence) for 9 h, and then its co-localization with membrane rafts was examined using fluorescein isothiocyanate-labeled CTx B subunit (green fluorescence for rafts). Areas of co-localization between membrane rafts and PTE-edelfosine in the merge panel are cyan. Data are representative of four independent experiments. Bar, 10 µm. (C) Higher magnifications of Jurkat cells treated as in B are shown. Data are representative of four independent experiments. Bar, 5 µm. (D, E) Jurkat cells were pretreated in the absence or presence of 2.5 mg/ml MCD for 30 min, and then drug uptake was determined after incubation with 10 µM [^3^H]edelfosine (EDLF) for 1 h (D), and apoptosis was analyzed by flow cytometry following incubation with 10 µM edelfosine for 24 h (E). Data are means±SE of three independent determinations.

Edelfosine itself accumulated in lipid rafts, as assessed by the presence of [^3^H]edelfosine in isolated rafts from Jurkat cells (Figure 7A). Furthermore, the fluorescent analogue PTE-edelfosine (all-[*E*]-1-*O*-[15′-phenylpentadeca-8′,10′,12′,14′-tetraenyl]-2-*O*-methyl-*rac*-glycero-3-phosphocholine), which behaves similarly to the parental drug [4], [19], co-localized with rafts forming edelfosine-rich lipid raft clusters in leukemic T-cells (Figure 7B). A higher magnification of the images showed a good co-localization between the green staining of the raft marker fluorescein isothiocyanate-labeled CTx B subunit and PTE-edelfosine (Figure 7C). Figures 7B and 7C also show the clustering and capping of lipid rafts induced by edelfosine treatment. Rafts as well as PTE-edelfosine were mainly concentrated in dense patches in one or two poles of the Jurkat cell (Figure 7B and 7C).

Preincubation with parental edelfosine prevented labeling of cells with the fluorescent analogue (data not shown). Disruption of lipid rafts with methyl-β-cyclodextrin (MCD), which interferes with protein association to rafts by cholesterol depletion [1], [27], inhibited drug uptake (Figure 7D), DISC formation (data not shown) and apoptosis (Figure 7E). No patchy localization of the edelfosine fluorescent analogue was observed at the cell surface of MCD-treated cells (data not shown). These results square with our previous observation that lipid raft disruption abrogated edelfosine-induced formation of Fas/CD95 clusters [1], [4]. Drug uptake was monitored after exhaustive washing of cells with bovine serum albumin (BSA), for which edelfosine shows a high binding capacity [28], [29]. Thus, incorporated edelfosine seems to be accumulated in the inner leaflet of the plasma membrane in order to be inaccessible to extracellularly added albumin.

## Discussion

**Figure 8:**
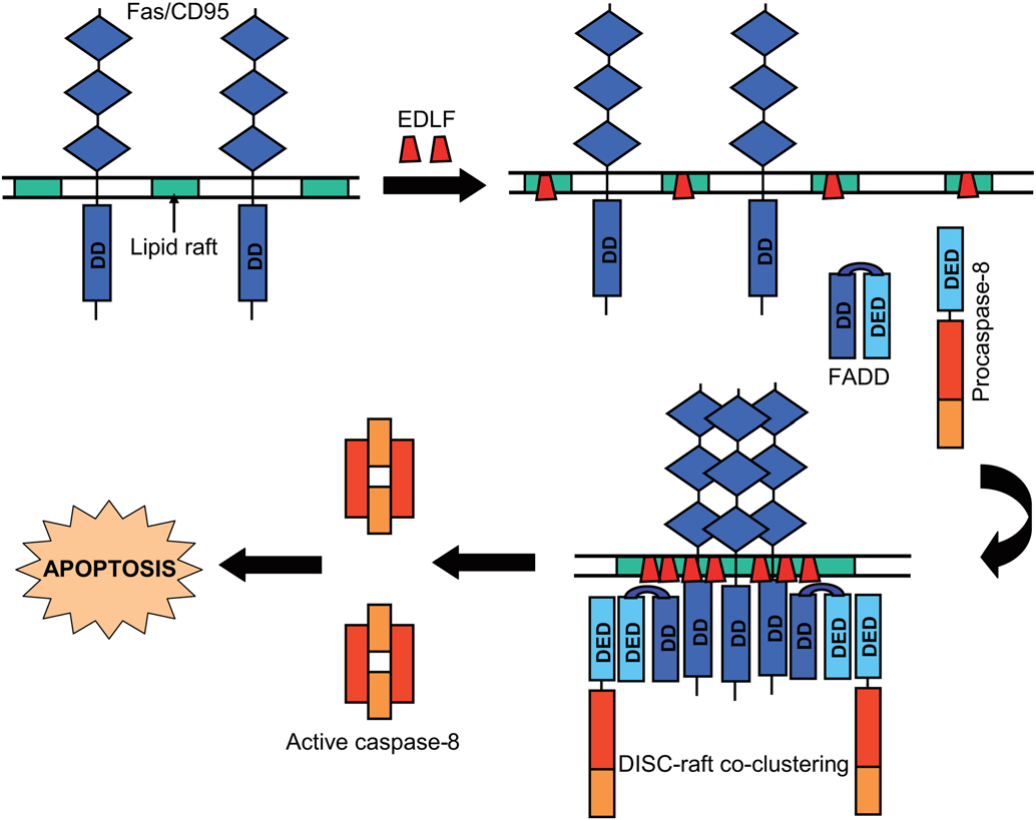
Schematic model for the involvement of DISC and lipid rafts in edelfosine-induced apoptosis in Jurkat cells. This diagram portrays a currently plausible mechanism for the role of DISC recruitment in membrane rafts in drug-induced apoptosis based on the results reported in this work. Initially, Fas/CD95 death receptor is not located at the membrane raft regions of plasma membrane. Incubation of Jurkat cells with edelfosine (EDLF) leads to its accumulation in membrane rafts, inducing raft clustering and recruitment of Fas/CD95 into lipid rafts. This translocation and concentration of Fas/CD95 in rafts brings together FADD and procaspase-8, forming the DISC, through protein-protein homotypic interactions between their respective death domains (DD) and death effector domains (DED). Thus, lipid raft clusters act as scaffolds where DISC is concentrated, hence achieving caspase-8 activation and eventually apoptosis. These DISC-raft co-clusters would behave as a supramolecular and physical entity crucial for the death receptor-mediated regulation of apoptosis.

Our data demonstrate that the synthetic alkyl-lysophospholipid analogue edelfosine targets lipid rafts in Jurkat leukemic T-cells, and that intact rafts are crucial for both drug uptake and drug-induced apoptosis, serving as scaffolds for DISC recruitment. The present results extend and complement our earlier findings on the role of CD95 signaling and lipid rafts in the triggering of apoptosis, and lead to the scheme depicted in Figure 8, as a novel framework in cancer therapy. Here we have shown, by using different experimental approaches, the formation and role of co-clusters of DISC and membrane rafts in the triggering of apoptosis during edelfosine antileukemic action. Crucial to this process is the accumulation of edelfosine in lipid rafts, which is ensued by the reorganization of membrane raft protein and lipid composition [4], [7], [30] that leads to the recruitment of DISC in rafts. The aggregation of DISC in membrane rafts would favor caspase-8 activation, and hence apoptosis, as the basal activity of procaspase-8 is put in function by proximity [31]. Thus, the concentration of DISC in a rather small region of the plasma membrane facilitates caspase-8 activation that eventually leads to apoptosis. Our present findings indicate that membrane rafts serve, in addition to generating high local concentrations of Fas/CD95, as platforms for coupling adapter (FADD) and effector (caspase-8) proteins required for Fas/CD95 signaling. This is of particular importance in Fas/CD95-mediated signal transduction as the initial signaling events depend on protein-protein interactions [1], [4], [8]. We have previously shown that edelfosine is taken up preferentially by tumor cells [12], [16], and trigger a Fas/CD95-mediated apoptotic response from within the cell independently of the ligand FasL/CD95L [4]. Taken together, these data suggest that formation of DISC-raft clusters can be pharmacologically modulated with promising therapeutic prospects in cancer therapy.

Recent evidence shows that the novel antitumor drugs aplidin and perifosine also induce translocation of Fas/CD95 and downstream signaling molecules into lipid rafts in leukemia cells [5], [7], [32]. In addition, cis-platin and resveratrol have been reported to elicit co-clustering of Fas/CD95 and rafts in solid tumors [21], [33]. Thus, there is an increasing evidence suggesting that Fas/CD95 translocation into lipid rafts represents a mechanism of action for anticancer drugs. The data reported here demonstrate the formation of DISC-enriched raft aggregates as a linchpin from which apoptosis is triggered. On these grounds, we postulate that co-aggregation of lipid rafts and Fas/CD95-DISC is a new framework and paradigm in anticancer therapy. Interestingly, edelfosine and aplidin, two anticancer drugs that promote recruitment of Fas/CD95 in lipid rafts, accumulate in death receptor-rich rafts [1], [4], [5]. Likewise, perifosine also binds to lipid rafts [34] and promotes Fas/CD95 translocation in rafts [7]. Taken together, these results establish membrane rafts as an attractive target in cancer therapy, and edelfosine antitumor action as a paradigm of this novel raft/death receptor-mediated mechanism of action. This translocation of Fas/CD95-DISC into raft clusters, leading to Fas/CD95-mediated apoptosis independently of FasL/CD95L, provides a new molecular insight into leukemia chemotherapy and in the triggering of death receptor-mediated apoptosis.

## Materials and Methods

### Cell culture

The human acute T-cell leukemia Jurkat cell line was grown in RPMI-1640 culture medium supplemented with 10% heat-inactivated fetal bovine serum (FBS), 2 mM L-glutamine, 100 U/ml penicillin, and 100 µg/ml streptomycin at 37°C in humidified 95% air and 5% CO_2_. The Jurkat-FADD-DN cell line, provided by M.L. Schmitz (Justus-Liebig-University, Giessen, Germany), is a Jurkat-derived clone stably transfected with a pcDNA3 expression vector encoding a dominant-negative form of the FADD protein [25]. This Jurkat-FADD-DN cell line constitutively expresses FADD-DN, thus blocking Fas/CD95 downstream signaling [25], and was maintained in complete medium containing 200 µg/ml G418. Jurkat cells were also transfected with control pcDNA3 empty vector using Lipofectamine 2000 (Invitrogen/Life Technologies, Carlsbad, CA) according to the manufacturer's instructions.

### Apoptosis assay by flow cytometry

Quantitation of apoptotic cells, following treatment with edelfosine (INKEYSA, Barcelona, Spain; APOINTECH, Salamanca, Spain), was calculated by flow cytometry as the percentage of cells in the sub-G_1_ region (hypodiploidy) in cell cycle analysis as previously described [17].

### TUNEL assay

Apoptosis was also analyzed *in situ* by the TUNEL technique using the Fluorescein Apoptosis Detection System (Promega, Madison, WI), according to the manufacturer's instructions. Cells were fixed on microscope slides, permeabilized with 0.2% Triton X-100, stained for fragmented DNA using the above kit, and then propidium iodide was added for 15 min to stain both apoptotic and non-apoptotic cells as previously described [16], [35]. Thus, propidium iodide stains apoptotic and non-apoptotic cells in red, whereas fluoresecin-12-dUTP is incorporated at the 3′-OH ends of fragmented DNA [26], resulting in localized green fluorescence within the nucleus of apoptotic cells. Samples were analyzed with a Zeiss LSM 510 laser scan confocal microscope.

### Edelfosine uptake

Drug uptake was measured as described previously [12] after incubating cells (10^6^) with 10 nmol [^3^H]edelfosine (10 µM) for 1 h in RPMI-1640/10% FBS, and subsequent exhaustive washing (six times) with PBS+2% BSA. [^3^H]edelfosine (specific activity, 42 Ci/mmol) was synthesized by tritiation of the 9-octadecenyl derivative (Amersham Buchler, Braunschweig, Germany).

### Edelfosine localization by fluorescence microscopy

Jurkat cells were treated for 9 h with 10 µM fluorescent analog PTE-edelfosine, provided by A.U. Acuña and F. Amat-Guerri (Consejo Superior de Investigaciones Científicas, Madrid, Spain), which behaves similarly to the parental compound [4], [19], and then incubated with the raft marker fluorescein isothiocyanate-CTx B subunit to label lipid rafts as described previously [1], [4]. Co-localization of the distinct fluorochromes was analyzed, by excitation of the corresponding fluorochromes in the same section of samples, using a fluorescence microscope (Axioplan 2; Carl Zeiss MicroImaging, Inc.) and a digital camera (ORCA-ER-1394; Hamamatsu).

### Lipid raft and DISC visualization by electron microscopy

Jurkat cells in culture medium were fixed 1∶1 (v/v) with 4% paraformaldehyde+0.4% glutaraldehyde pH 7.4 in 0.2 M PHEM buffer (60 mM PIPES, 25 mM HEPES, 2 mM MgCl_2_, 10 mM EGTA) for 30 min at room temperature. Cells were washed in PHEM and treated with 40 mM glycine. Lipid rafts were detected by incubation for 1 h at room temperature with cholera toxin (CTx) B subunit conjugated to biotin (Invitrogen, San Diego, CA) diluted 1∶10 in blocking solution (PHEM-2% BSA). Cells were washed with PBS and incubated for 1 h at room temperature with 6-nm gold particles conjugated to streptavidin (Electron Microscopy Sciences, Hatfield, PA), diluted 1∶40 in PBS. Cells were then fixed overnight in 2% glutaraldehyde in PHEM buffer. Samples were treated for electron microscopy by postfixing the cells for 1 h in 1% osmium tetroxide in phosphate buffer pH 7.4 containing 1% CaCl_2_. Cells were then embedded in Durcupan resin (Fluka/Sigma Aldrich, Buchs, Switzerland). Ultrathin sections were mounted on formvar-coated nickel grids and stained with 2% uranyl acetate for 20 min and lead citrate for 2 min. Grids were examined on a Zeiss EM 900 at 80 kV accelerating voltage. Cells without lipid raft labeling were used as negative controls, showing no staining of the samples.

Cells with labeled lipid rafts were fixed in 2% paraformaldehyde plus 0.5% glutaraldehide in 0.1 M PHEM buffer overnight and embedded in London Resin White (London Resin Company, Berkshire, UK). Ultrathin sections were mounted on formvar-coated nickel grids. Visualization of lipid rafts was processed as above using 6-nm gold particles conjugated to streptavidin. For immunolabeling, grids were floated at room temperature on drops containing 40 mM glycine in TBST (50 mM Tris-HCl, pH 8.0, 150 mM NaCl, 0.05% Tween) for 30 min to quench unreacted aldehydes. After blocking with 2% BSA in TBST for 30 min, grids were incubated for 1 h with goat anti-procaspase-8 antibody (C20) (Santa Cruz Biotechnology, Santa Cruz, CA) (dilution 1∶10 in blocking solution), and then with anti-goat IgG conjugated with 20-nm gold particles (Electron Microscopy Sciences) (dilution 1∶50 in blocking solution). The grids were put successively through PBS, 1% glutaraldehyde in PBS (10 min), PBS, PBS glycine, 2% BSA-TBST. For double immunolabeling, Fas/CD95 and FADD proteins were detected following 1 h incubation each with specific anti-Fas/CD95 (C-20) rabbit antibody (Santa Cruz Biotechnology) and anti-FADD (clone-1) mouse monoclonal antibody (BD Transduction Laboratories, Lexington, KY) (dilution 1∶10 in blocking solution). Grids were incubated with a mixture of anti-mouse IgG coupled to 15 nm gold particles and anti-rabbit IgG linked to 10 nm gold particles, both diluted 1∶50 in blocking solution. In order to reduce background, NaCl concentration was elevated from 2.5 mM to 2.5 M in 4 changes of 6 min each and then switched to ultrapure water. Grids were counterstained with 2% uranyl acetate for 20 min in dark and examined in a Zeiss EM 900. Negative controls were prepared by replacing the primary antibody with a nonrelevant antibody, showing no staining of the samples.

### Transfection of Jurkat cells with Fas/CD95 shRNA

Jurkat cells were successfully transfected with a negative control neomycin vector containing a scrambled sequence (5′-GGAATCTCATTCGATGCATAC-3′) and four pre-designed Fas/CD95 shRNA vectors (SureSilencing shRNA, SuperArray Bioscience Corporation, Frederick, MD) containing 4 target sequences (1: 5′-GCTGGAGTCATGACACTAAGT-3′; 2: 5′-GAAGCGTATGACACATTGATT-3′; 3: 5′-TTGGAAGGCCTGCATCATGAT-3′; 4: 5′-AACCAAGGTTCTCATGAATCT-3′), using Lipofectamine 2000 (Invitrogen), according to the manufacturer's instructions. Jurkat cells were plated in 24-well plates at 3×10^4^ cells/cm^2^. shRNA plasmid (0.5 µg) was mixed with 50 µl Opti-MEM I reduced-serum medium (Life Technologuies, Gaithersburg, MD) for 10 min at room temperature. Lipofectamine 2000 (1 µl) was diluted in 50 µl Opti-MEM medium and mixed gently with the shRNA solution. After a 20-min incubation at room temperature, mixture was added to the cells in a final volume of 0.5 ml/well. Transfected cells were cultured at 37°C in humidified 95% air and 5% CO_2_ for 48 h, and then neomycin-resistant clones were selected using G418 (1 mg/ml). Silencing of Fas/CD95 expression was confirmed by flow cytometry and Western blotting. Only one (target sequence 1) of the four Fas/CD95 shRNA sequences successfully inhibited Fas/CD95 expression (about 65% inhibition).

### Immunofluorescence flow cytometry

Cell surface expression of Fas/CD95 death receptor was analyzed by flow cytometry in 4×10^5^ cells as described previously [16] in a Becton Dickinson FACSCalibur™ flow cytometer using an anti-Fas/CD95 SM1/1 monoclonal antibody (Bender MedSystems, Vienna, Austria). P3X63 myeloma culture supernatant, provided by F. Sánchez-Madrid (Hospital de La Princesa, Madrid, Spain), was used as a negative control.

### Lipid raft isolation

Lipid rafts were isolated from 8×10^7^ cells by nonionic detergent lysis and centrifugation on discontinuous sucrose gradients as described [1], [4]. Twelve fractions (1-ml) were collected from the top of the gradient and 20 µl of each fraction were subjected to SDS-PAGE, immunoblotting and enhanced chemiluminescence detection. Location of GM1-containing lipid rafts was determined using CTx B subunit conjugated to horseradish peroxidase (Sigma). Pools of fractions 3–5 (enriched in lipid rafts) and fractions 10–12 (largely deficient in lipid rafts) from these sucrose gradients led to the raft and non-raft fractions, respectively, which were subsequently subjected to Western blotting. Proteins were identified using specific antibodies: anti-48-kD Fas/CD95 (C-20) rabbit polyclonal antibody (Santa Cruz Biotechnology), anti-29-kD FADD (clone-1) (BD Transduction Laboratories) monoclonal antibody, and anti-55-kDa procaspase-8 (Ab-3) monoclonal antibody (Oncogene Research Products, Cambridge, MA).

### Co-immunoprecipitation assays

Fractions 3–5 from the lipid raft isolation sucrose gradient were mixed and incubated with lysis buffer (20 mM Tris-HCl, pH 7.5, 100 mM KCl, 0.9% Triton X-100, 10% glycerol, 2 mM Na_3_VO_4_, 2 mM PMSF). Lysates were precleared with protein A-Sepharose, and immunoprecipitated with anti-Fas/CD95 (C-20) rabbit polyclonal antibody precoupled to protein A-Sepharose as previously described [7], [36]. Samples were subjected to SDS-PAGE and immunoblotted with specific antibodies against FADD and procaspase-8. Membrane raft pools were also immunoprecipitated with P3X63 myeloma supernatant as a negative control showing no signal.

### Cholesterol depletion

For cholesterol depletion, 2.5×10^5^ cells/ml were pretreated with 2.5 mg/ml MCD for 30 min at 37°C in serum-free medium. Cells were then washed three times with PBS and resuspended in complete culture medium before edelfosine addition.

## References

[1] Gajate C, Mollinedo F (2001). The antitumor ether lipid ET-18-OCH_3_ induces apoptosis through translocation and capping of Fas/CD95 into membrane rafts in human leukemic cells.. Blood.

[2] Hueber AO, Bernard AM, Herincs Z, Couzinet A, He HT (2002). An essential role for membrane rafts in the initiation of Fas/CD95-triggered cell death in mouse thymocytes.. EMBO Rep.

[3] Scheel-Toellner D, Wang K, Singh R, Majeed S, Raza K (2002). The death-inducing signalling complex is recruited to lipid rafts in Fas-induced apoptosis.. Biochem Biophys Res Commun.

[4] Gajate C, Del Canto-Janez E, Acuna AU, Amat-Guerri F, Geijo E (2004). Intracellular triggering of Fas aggregation and recruitment of apoptotic molecules into Fas-enriched rafts in selective tumor cell apoptosis.. J Exp Med.

[5] Gajate C, Mollinedo F (2005). Cytoskeleton-mediated death receptor and ligand concentration in lipid rafts forms apoptosis-promoting clusters in cancer chemotherapy.. J Biol Chem.

[6] Mollinedo F, Gajate C (2006). Fas/CD95 death receptor and lipid rafts: New targets for apoptosis-directed cancer therapy.. Drug Resist Updat.

[7] Gajate C, Mollinedo F (2007). Edelfosine and perifosine induce selective apoptosis in multiple myeloma by recruitment of death receptors and downstream signaling molecules into lipid rafts.. Blood.

[8] Mollinedo F, Gajate C, Wajant H (2006). FasL-independent activation of Fas.. Fas Signaling.

[9] Ogasawara J, Watanabe-Fukunaga R, Adachi M, Matsuzawa A, Kasugai T (1993). Lethal effect of the anti-Fas antibody in mice.. Nature.

[10] Tanaka M, Suda T, Yatomi T, Nakamura N, Nagata S (1997). Lethal effect of recombinant human Fas ligand in mice pretreated with Propionibacterium acnes.. J Immunol.

[11] Mollinedo F (2007). Antitumor ether lipids: proapoptotic agents with multiple therapeutic indications.. Expert Opin Ther Patents.

[12] Mollinedo F, Fernandez-Luna JL, Gajate C, Martin-Martin B, Benito A (1997). Selective induction of apoptosis in cancer cells by the ether lipid ET-18-OCH_3_ (Edelfosine): molecular structure requirements, cellular uptake, and protection by Bcl-2 and Bcl-X_L_.. Cancer Res.

[13] Mollinedo F, Gajate C, Modolell M (1994). The ether lipid 1-octadecyl-2-methyl-*rac*-glycero-3-phosphocholine induces expression of *fos* and *jun* proto-oncogenes and activates AP-1 transcription factor in human leukaemic cells.. Biochem J.

[14] Gajate C, Santos-Beneit A, Modolell M, Mollinedo F (1998). Involvement of c-Jun NH_2_-terminal kinase activation and c-Jun in the induction of apoptosis by the ether phospholipid 1-*O*-octadecyl-2-*O*-methyl-*rac*-glycero-3-phosphocholine.. Mol Pharmacol.

[15] Ruiter GA, Zerp SF, Bartelink H, van Blitterswijk WJ, Verheij M (1999). Alkyl-lysophospholipids activate the SAPK/JNK pathway and enhance radiation-induced apoptosis.. Cancer Res.

[16] Gajate C, Fonteriz RI, Cabaner C, Alvarez-Noves G, Alvarez-Rodriguez Y (2000). Intracellular triggering of Fas, independently of FasL, as a new mechanism of antitumor ether lipid-induced apoptosis.. Int J Cancer.

[17] Gajate C, Santos-Beneit AM, Macho A, Lazaro M, Hernandez-De Rojas A (2000). Involvement of mitochondria and caspase-3 in ET-18-OCH_3_-induced apoptosis of human leukemic cells.. Int J Cancer.

[18] Nieto-Miguel T, Fonteriz RI, Vay L, Gajate C, Lopez-Hernandez S (2007). Endoplasmic reticulum stress in the proapoptotic action of edelfosine in solid tumor cells.. Cancer Res.

[19] Nieto-Miguel T, Gajate C, Mollinedo F (2006). Differential targets and subcellular localization of antitumor alkyl-lysophospholipid in leukemic *versus* solid tumor cells.. J Biol Chem.

[20] Peter ME, Krammer PH (2003). The CD95(APO-1/Fas) DISC and beyond.. Cell Death Differ.

[21] Delmas D, Rebe C, Lacour S, Filomenko R, Athias A (2003). Resveratrol-induced apoptosis is associated with Fas redistribution in the rafts and the formation of a death-inducing signaling complex in colon cancer cells.. J Biol Chem.

[22] Schon A, Freire E (1989). Thermodynamics of intersubunit interactions in cholera toxin upon binding to the oligosaccharide portion of its cell surface receptor, ganglioside GM1.. Biochemistry.

[23] Cheng PC, Dykstra ML, Mitchell RN, Pierce SK (1999). A role for lipid rafts in B cell antigen receptor signaling and antigen targeting.. J Exp Med.

[24] Harder T, Scheiffele P, Verkade P, Simons K (1998). Lipid domain structure of the plasma membrane revealed by patching of membrane components.. J Cell Biol.

[25] Hofmann TG, Moller A, Hehner SP, Welsch D, Droge W (2001). CD95-induced JNK activation signals are transmitted by the death-inducing signaling complex (DISC), but not by Daxx.. Int J Cancer.

[26] Gavrieli Y, Sherman Y, Ben-Sasson SA (1992). Identification of programmed cell death in situ via specific labeling of nuclear DNA fragmentation.. J Cell Biol.

[27] Christian AE, Haynes MP, Phillips MC, Rothblat GH (1997). Use of cyclodextrins for manipulating cellular cholesterol content.. J Lipid Res.

[28] Kelley EE, Modest EJ, Burns CP (1993). Unidirectional membrane uptake of the ether lipid antineoplastic agent edelfosine by L1210 cells.. Biochem Pharmacol.

[29] Tsutsumi T, Tokumura A, Kitazawa S (1998). Undifferentiated HL-60 cells internalize an antitumor alkyl ether phospholipid more rapidly than resistant K562 cells.. Biochim Biophys Acta.

[30] Zaremberg V, Gajate C, Cacharro LM, Mollinedo F, McMaster CR (2005). Cytotoxicity of an anti-cancer lysophospholipid through selective modification of lipid raft composition.. J Biol Chem.

[31] Muzio M, Stockwell BR, Stennicke HR, Salvesen GS, Dixit VM (1998). An induced proximity model for caspase-8 activation.. J Biol Chem.

[32] Gajate C, An F, Mollinedo F (2003). Rapid and selective apoptosis in human leukemic cells induced by Aplidine through a Fas/CD95- and mitochondrial-mediated mechanism.. Clin Cancer Res.

[33] Lacour S, Hammann A, Grazide S, Lagadic-Gossmann D, Athias A (2004). Cisplatin-induced CD95 redistribution into membrane lipid rafts of HT29 human colon cancer cells.. Cancer Res.

[34] van der Luit AH, Vink SR, Klarenbeek JB, Perrissoud D, Solary E (2007). A new class of anticancer alkylphospholipids uses lipid rafts as membrane gateways to induce apoptosis in lymphoma cells.. Mol Cancer Ther.

[35] Cabaner C, Gajate C, Macho A, Munoz E, Modolell M (1999). Induction of apoptosis in human mitogen-activated peripheral blood T-lymphocytes by the ether phospholipid ET-18-OCH_3_: involvement of the Fas receptor/ligand system.. Br J Pharmacol.

[36] Mollinedo F, Martin-Martin B, Calafat J, Nabokina SM, Lazo PA (2003). Role of vesicle-associated membrane protein-2, through Q-soluble *N*-ethylmaleimide-sensitive factor attachment protein receptor/R-soluble *N*-ethylmaleimide-sensitive factor attachment protein receptor interaction, in the exocytosis of specific and tertiary granules of human neutrophils.. J Immunol.

